# Mussel-Inspired Magnetic Dissolving Pulp Fibers Toward the Adsorption and Degradation of Organic Dyes

**DOI:** 10.3389/fchem.2022.840133

**Published:** 2022-03-15

**Authors:** Jiawei Yang, Shengchang Lu, Hui Wu, Huichao Hu, Qingxian Miao, Liulian Huang, Lihui Chen, Yonghao Ni

**Affiliations:** ^1^ College of Material Engineering, Fujian Agriculture and Forestry University, Fuzhou, China; ^2^ National Forestry and Grassland Administration Key Laboratory of Plant Fiber Functional Materials, Fuzhou, China; ^3^ School of Forestry, Henan Agricultural University, Zhengzhou, China; ^4^ Department of Chemical Engineering, Limerick Pulp and Paper Centre, University of New Brunswick, Fredericton, NB, Canada

**Keywords:** magnetic dissolving, absorbance, degradation, organic dye, synthetic method

## Abstract

In this work, a simple synthetic method was used to prepare a new type of magnetic dissolving pulp (MDP) @polydopamine (PDA) fibers. The hydroxyl groups of the fibers were converted into carboxyl groups after succinylation. Fe_3_O_4_ nanoparticles were grown *in situ* on the fibers. The prepared MDP@PDA fibers have catalytic reduction efficiency and adsorption performance for methylene blue organic dyes, and it has been thoroughly tested under various pH conditions. Fe_3_O_4_@PDA fibers have high reusability, are easy to separate, and regenerate quickly. The catalytic and adsorption efficiency barely decreases after repeated use. The surface of dissolving pulp fibers with a functionalized multifunctional PDA coating is used to create multifunctional catalysts and adsorbent materials. This study presents a very useful and convenient method for the synthesis and adjustment of MDP@PDA fibers, which have a wide range of potential applications in catalysis and wastewater treatment.

## Introduction

Water pollution by organic contaminants has become a serious environmental issue and received significant attention (Wang et al., 2009; Xie et al., 2014). Among many organic pollutants, it is critical to address pollution issues with organic dyes. Water contamination by dyes from various industries, such as textiles, leather, and dyestuffs, has gotten more attention because the dyes in these industrial wastewaters are toxic, carcinogenic, and nonbiodegradable (Yuanyuan Liu et al., 2015; Cho et al., 2015). As a result, a variety of technologies, including adsorption (Hossain et al., 2021), photocatalytic degradation, chemical reduction or chemical oxidation, membrane filtration, flocculation, and electro-oxidation, have been used to remove these contaminants (Panizza and Cerisola, 2009; Ali, 2012). Adsorption is one of the most effective methods to treat organic dyes, and it is widely used because of its relatively low cost and easy operation. Various adsorbents have been designed to remove dyes in water and have considerable adsorption capacities, such as activated carbon (Tang et al., 2017; Zhang et al., 2019; Soares et al., 2021), functional carbon materials (Magno et al., 2020; Xing et al., 2020; Zhang et al., 2020), composite hydrogel (Srivastava and Roy Choudhury, 2021), and metal oxide (Rastogi et al., 2017; Xu et al., 2020; Şerban and Enesca, 2020). Among these methods, chemistry reduction has gained popularity because of its ease of use, high efficiency, clean processing, and low cost. The chemical reduction of various dyes with NaBH_4_ is an important, safe, and environmentally friendly alternative. This process is thermodynamically advantageous in the absence of a catalyst, but kinetics is difficult. Because the redox potential difference between the electron donor and acceptor hinders electron transfer, the electron transfer step is dynamic. As a result, an efficient catalyst with a moderate oxidation–reduction potential can serve as an electron shuttle, facilitating electron transfer.

Redox mediators (RMs) play a key role in improving the performance of electron-receiving priority pollutants. As RMs can be reversibly oxidized and reduced (Coletti et al., 2012; Lentini et al., 2014), they can be used as electron transfer means in multiple redox reactions, increasing the reaction rate by one or more orders of magnitude. Because water-soluble RMs that are washed away with wastewater are typically used, RMs must be added continuously, raising the processing cost. Furthermore, the RMs in the effluent will cause secondary pollution. As a result, the ideal RM for catalytic pigment degradation should promote sewage purification while minimizing secondary pollution. RM immobilized on a suitable carrier shows great potential as a catalyst in these processes (Du et al., 2017).

Inspired by the marine mussels, which have the unique wet adhesion capability, polydopamine (PDA) has attracted strong interest as a biomimetic polymer (Tang et al., 2021) and a universal surface modification agent for various materials with a broad range of applications (Zeng et al., 2010; Bandara et al., 2012; Wang et al., 2018; Lin et al., 2019; Lin et al., 2020; Wang et al., 2020; Xiang et al., 2020; Lu et al., 2021). PDA-functionalized hybrid materials were recently developed as a novel nanostructured adsorbent for dye removal. In the field of wastewater treatment, Chen et al. developed a PDA modified cyclodextrin polymer adsorbent (Chen et al., 2020). Mu et al. described a chitosan/PDA@C@magnetic fly ash adsorbent bead for Ag (I) ion adsorption in aqueous solutions (Mu et al., 2020). With the help of PDA catechol groups, these adsorbents demonstrated excellent cationic dye and metal ion adsorption (Xie et al., 2014). Dissolving pulp (DP) is a high-grade cellulose pulp (90%–98% cellulose content), with low hemicellulose, lignin, and resin content (Peng et al., 2012; Arce et al., 2020), which has been steadily increasing in popularity over the last decade (Llano et al., 2018; Liu et al., 2021). Cellulose is regarded as the most abundant and renewable natural resource on the earth and promising as adsorbents owing to the inherent porous structure. The functionalization of cellulose tends to lead to an even higher adsorption capacity for days (Kim et al., 2017; Li et al., 2018; Lu et al., 2018).

Herein, we present a facile synthetic method of *in situ* reductions to prepare MDP@PDA fibers, where PDA acts as a coating to adsorb the dyes (Lee et al., 2007). It is celebrated that under mild solution conditions, dopamine can automatically self-polymerize into PDA, which can be used as a binder and coating on various substrates, as inspired by the unique underwater adhesion properties of marine mussel foot proteins (Lee et al., 2007; Zeng et al., 2010; Bandara et al., 2012). MDP@PDA fibers are easy to separate in water because of its magnetism. Thus, a simple method for synthesizing novel MDP@PDA fibers with obviously fast adsorption ability for methylene blue (MB) at different pH values and easily separated from the water was developed in this work. The modified fibers have exceptional cyclic performance.

## Materials and Methods

### Materials

DP was procured from Fujian Qinshang Paper Industry Co., Ltd. Succinic anhydride, iron (Ⅱ) chloride (FeCl_2_), and ammonia solution (25–30% NH_3_) were obtained from Innochem. Aladdin provided dopamine hydrochloride, MB, pyridine, iron (III) chloride (FeCl_3_) hexahydrate, and sodium chloride (NaCl). Macklin and Xilong Scientific supplied sodium hydroxide and toluene, respectively. Ultrapure water was obtained using LabTower EDI 15 system (Thermo, USA) and was applied for all experiments.

### Succinylation of DP

The DP board was dispersed into a cotton shape with fiber dissociator, and a total of 0.5 g of the DPs was immersed in an aqueous solution of 10 wt% NaOH (50 mL) for 24 h. Then, the samples were repeatedly washed with absolute ethanol until a solution with neutral pH was obtained. After removing the moisture from the pulps in a vacuum drying oven (40°C), the DPs were used for further reaction. Pretreated DP (0.5 g, 9.1 mmol of active-OH groups (Roy et al., 2005)) was reacted with succinic anhydride (2.5 g, 25 mmol) at 90°C for 24 h in 10 mL of a mixed solvent containing toluene (35 mL) and pyridine. Then, the pulp samples were removed from the reaction and washed with toluene at least three times and soon afterward with acetone about five times before using a vacuum drying oven (40°C). Finally, the succinylation (SA)–DP was allowed to store in a desiccator until further analysis or functionalization.

### Preparation of Magnetic DP@PDA Fibers

The magnetic dissolving pulps (MDPs) were fabricated through the *in situ* synthesis of Fe_3_O_4_, located in the porous of carboxylated DPs (Xu et al., 2012). By stirring under nitrogen, the succinylated DPs (0.5 g) were added in a mixture solution of FeCl_2_ (2.7 g, 0.1 mol) and FeCl_3_ (0.63 g, 0.05 mol) (mole ratio FeCl_3_:FeCl_2_ = 2:1, and ultrapure water was 50 mL) in a 200 mL two-necked flask. Then, while stirring, an excessive ammonia solution (approximately 50 mL) was added and reacted for 1 h. The obtained reaction product was filtered and washed with a large amount of deionized water to remove any remaining ammonia and impurities before being freeze-dried. Afterward, a dopamine hydrochloride (0.25 mmol) solution dissolved in phosphate-buffered saline (pH 8.5, titrated with NaOH) was prepared (Park et al., 2020). Subsequently, the prepared MDP fibers (0.5 g) were mixed into the dopamine solution in a 200-mL single-neck flask and reacted on an on-air table for 8 h.

### Characterizations

Fourier transform infrared spectroscopy (FTIR) spectra of the dried DP, SA-DP, MDP@PDA, and MB adsorbed MDP@PDA were recorded on a Bruker VERTEX 70 FTIR spectrometer in the region of 400–4,000 cm^−1^. The surface morphologies of the synthesized MDP@PDA samples were achieved using a field emission scanning electron microscopy at a voltage of 5 kV. On a Kratos Axis Ultra spectrometer set to 30 eV, the X-ray photoelectron spectroscopy (XPS) spectra were recorded using Al Kα radiation. A vibrating sample magnetometer was used to measure the magnetic properties (VSM, 7,307; Lakeshore, USA). The DP and MDP@PDA fibers were subjected to thermogravimetric analysis using the synchronous thermal analyzer (Mettler TGA/DSC3+, Switzerland). Five milligrams of sample was placed in the crucible and heated from 30°C to 600°C at a rate of 10°C · min^−1^ under N_2_ flow. The experiment of Zeta potential was performed using a similar method as previous literature (Zhang et al., 2018); briefly, 0.1 g · L^−1^ DP and MDP@PDA fibers dispersion was analyzed by Malvern Zetasizer (NanoZS90X, United Kingdom).

### Adsorption Experiments

A 20 mg portion of MDP@PDA fibers was added into 10 mL MB aqueous solution (pH 6.2, 40 mg · L^−1^) under constant stirring at room temperature. To monitor the adsorption process, the effect of pH (3, 5, 7, 9) was investigated with 20 mg MDP@PDA and dye initial concentration of 40 mg · L^−1^, respectively; NaCl (0.05, 0.1, and 0.2 g · L^−1^) was used to evaluate the influences of electrolyte on dye removal efficiency; the UV-vis absorption spectra of MB at various pH and ion concentrations were recorded. The adsorbents were separated from the solution with an external magnet at the end of adsorption. Before the next desorption–adsorption cycle; the recycled adsorbents were washed with fresh NaBH_4_ aqueous solution (2 mL, 1.5 mmol · L^−1^) and then washed with ethanol and ultrapure water at least three times. The formula for calculating the decolorization ratio (*D*_color_ *R*, %) is as follows:

$$D_{\text{color}}\;R=\frac{C_{0}-C_{1}}{C_{0}}\times 100\%$$ (1)

where *C*_0_ and *C*_1_ are the dye concentrations (mg · L^−1^) in the initial reaction and postreaction solution, respectively.

### Catalytic Reduction Experiments

A 10-mg portion of MDP@PDA fibers was added to a 10 mL MB aqueous solution. Subsequently, 0.1 mL of fresh NaBH_4_ aqueous solution (0.1 mol · L^−1^) was injected into the solution under stirring. The blue color of MB gradually vanished by catalytic reduction, and the decolorization ratio was calculated by measuring the changes in the absorbance at 665 nm with a UV-vis spectrophotometer based on Equation 1.

## Results and Discussion

### Synthesis and Characterizations of Materials

**FIGURE 1:**
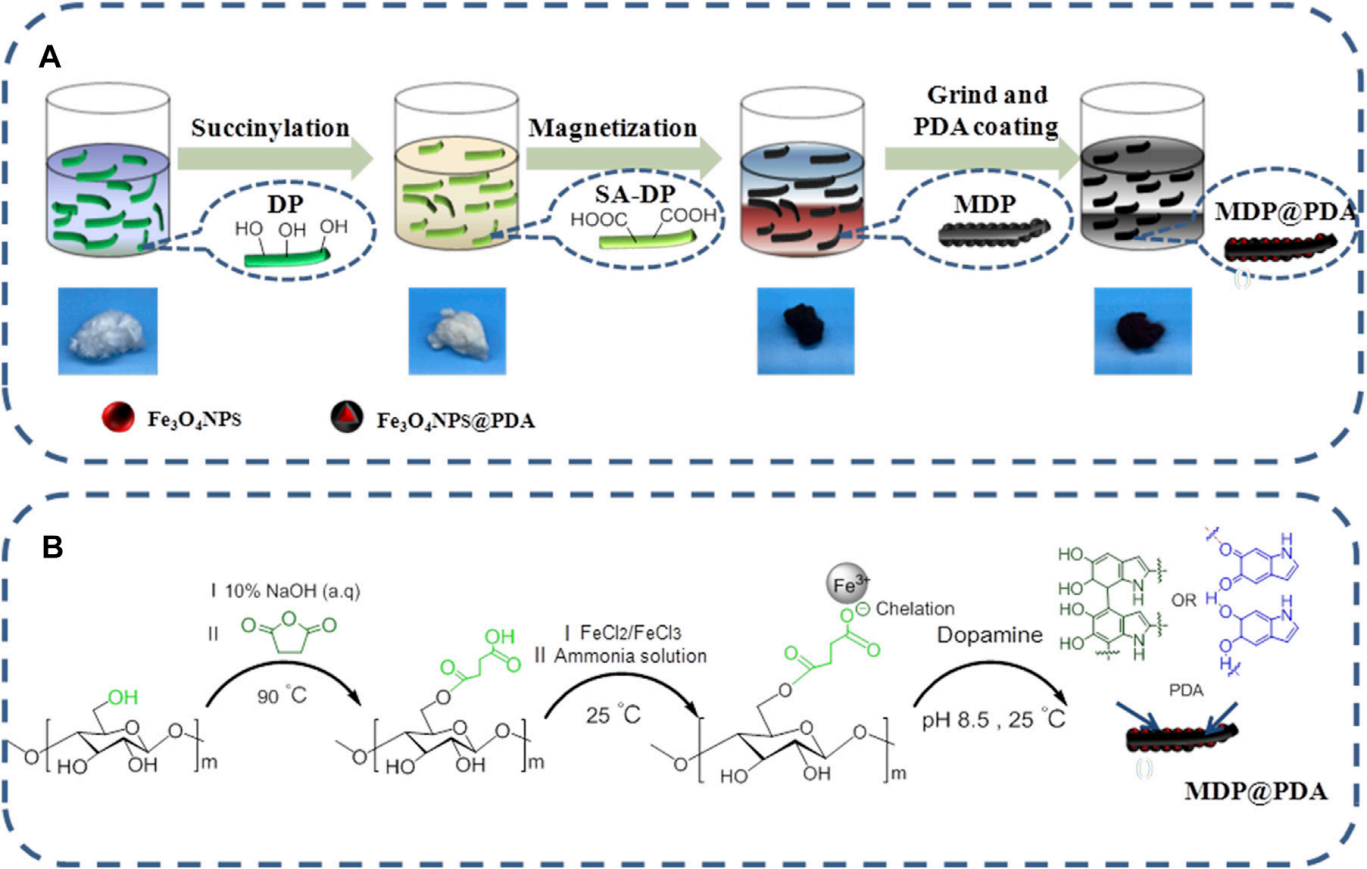
**(A)** Scheme of synthesis of MDP@PDA fibers. **(B)** The synthesis of MDP@PDA.

Our mussel-inspired strategy, as described in Figure 1, requests the adjunct of the dopamine molecules onto the DP fibers, which is composed of β-glucose units containing primary alcohol (-OH) groups, and we exploited these alcohol groups for functionalizing the finer surface by succinylation. The alcohol (-OH) groups of the glucose unit, on the other hand, are not readily available for functionalization because the majority of them form intermolecular hydrogen bonds with the attachment of alcohol (-OH) groups, leaving only a few available for surface reaction (Lee et al., 2015). After being succinylated, the DP fibers were treated with a 10% NaOH aqueous solution to disrupt the extensive hydrogen bonds between fibers by deprotonating the primary alcohol (-OH) groups of glucose units, and we can conclude that the alkali treatment of DP fibers was able to increase the degree of succinylation (Tankhiwale and Bajpai, 2008; Islam et al., 2018). Typically, SA-DP fibers were first synthesized through a succinic anhydride and used pyridine as anhydride catalyst in toluene solution, and the white fibers turned yellowish. Afterward, the SA-DP was added to the iron ion (the ratio of Fe^3+^ and Fe^2+^ is 2:1) mixed solution, and then the iron ions were reduced *in situ* by adding ammonia to make the fibers black and magnetic. Finally, the MDP fibers were blended into dopamine solution and adjusted the pH to 8.5 to make the dopamine self-aggregate on the fiber surface (Figure 1A); fibers still appeared black.

**FIGURE 2:**
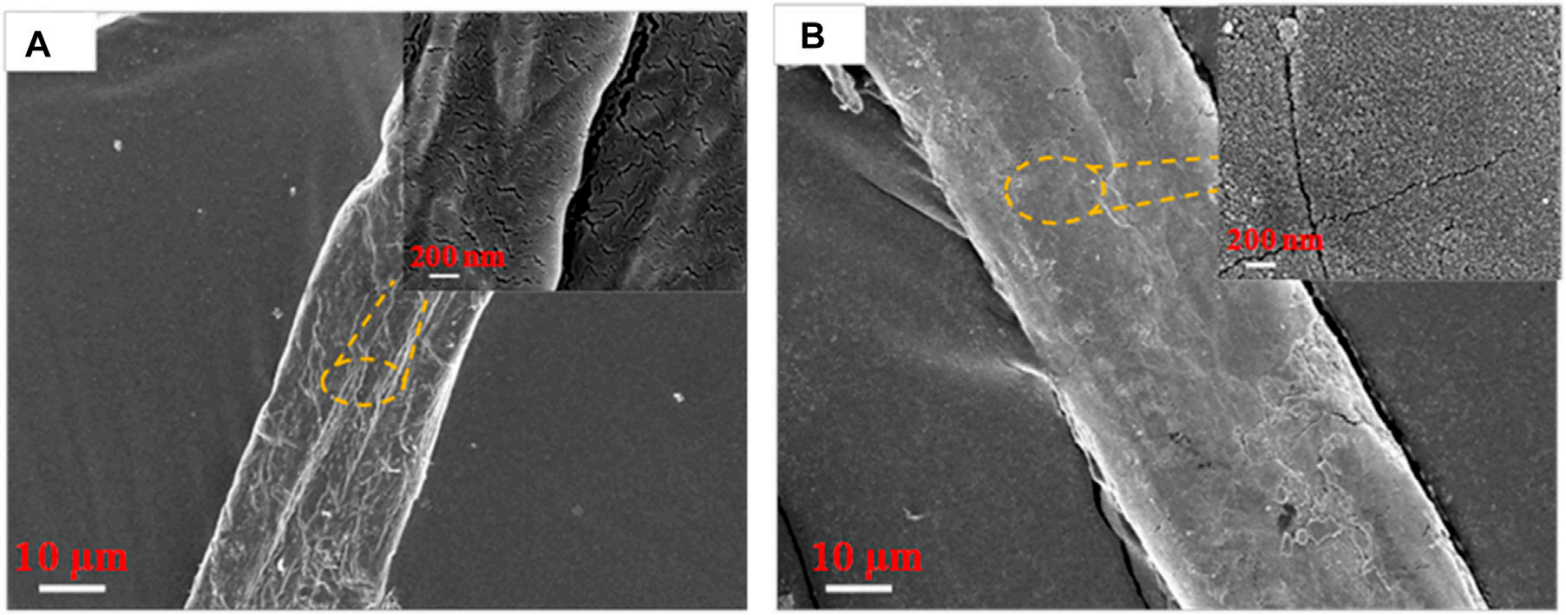
SEM images of fiber surface: **(A)** DP and **(B)** MDP@PDA fibers.

**FIGURE 3:**
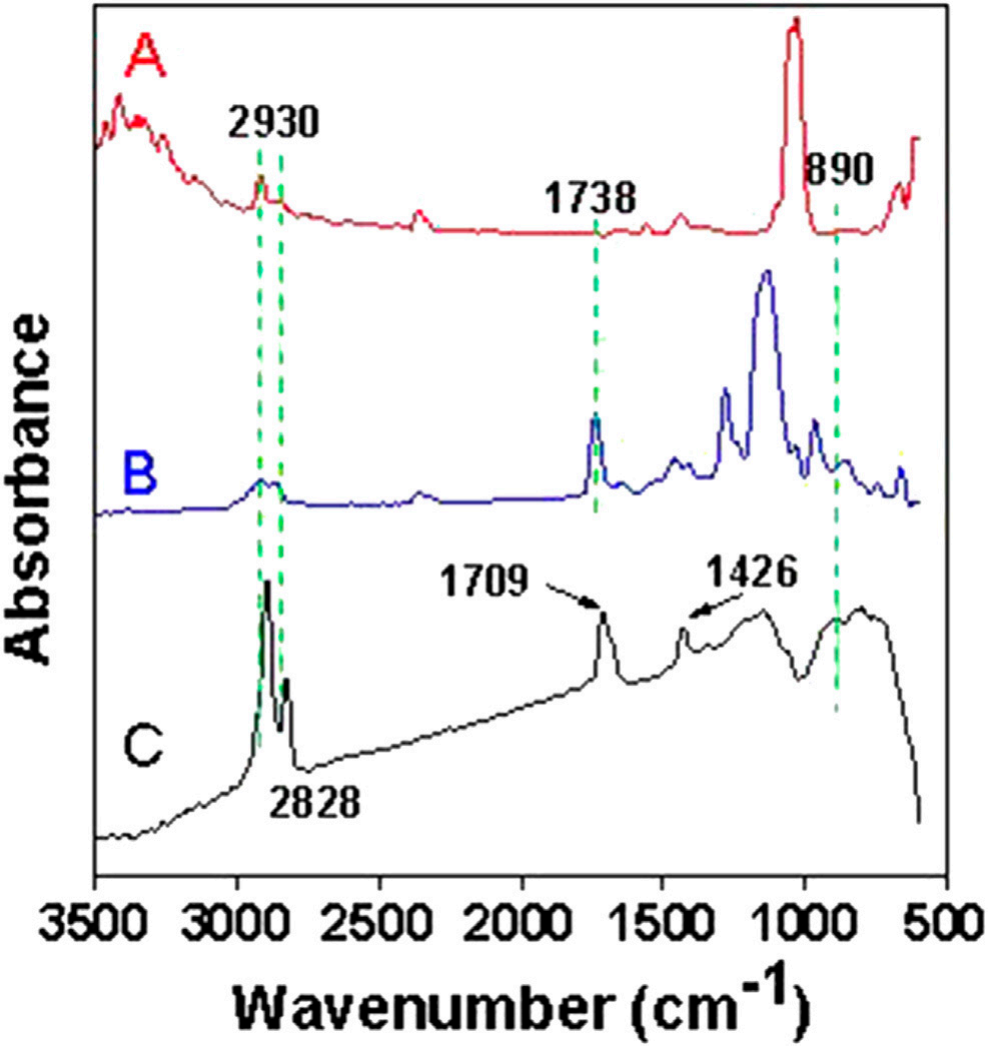
ATR-FTIR spectra of **(A)** DP, **(B)** SA-DP, and **(C)** MDP@PDA.

The microscopic morphology of DP and MDP@PDA was characterized by SEM to observe the fiber surface after the reaction. The results showed that, when compared with DP in Figure 2A, nanosized PDA particles are generated on the surface of MDP@PDA in Figure 2B, and a large amount of them agglomerates (Peng et al., 2012). Because of dopamine’s catechol and amino-functional structure, which can form covalent and noncovalent bond interactions with organic–inorganic surfaces, the PDA cross-linked layer is attached to the surface of fibers (Lee et al., 2006; Lee et al., 2007). To confirm the chemical structural changes of the DP after experiencing their successive reaction, infrared absorbance experiments were performed on DP, SA-DP, and MDP@PDA, The result of ATR-FTIR spectra is shown in Figure 3. In all ATR-FTIR spectra, the peaks 2,930 and 2,828 cm^−1^ are related to methylene asymmetry and symmetric stretching vibration (Otsuka et al., 2017), which explained the existence of cellulose backbone structure. In Figure 3B, compared with DP in Figure 3A, the absorbance observed at 1,738 cm^−1^ revealed the formation of carbonyl structures, especially the C=O (Ding et al., 2017). What’s more, the result showed in Supplementary Figure S2 indicated that MB was adsorbed onto the MDP@PDA fibres successfully.

**FIGURE 4:**
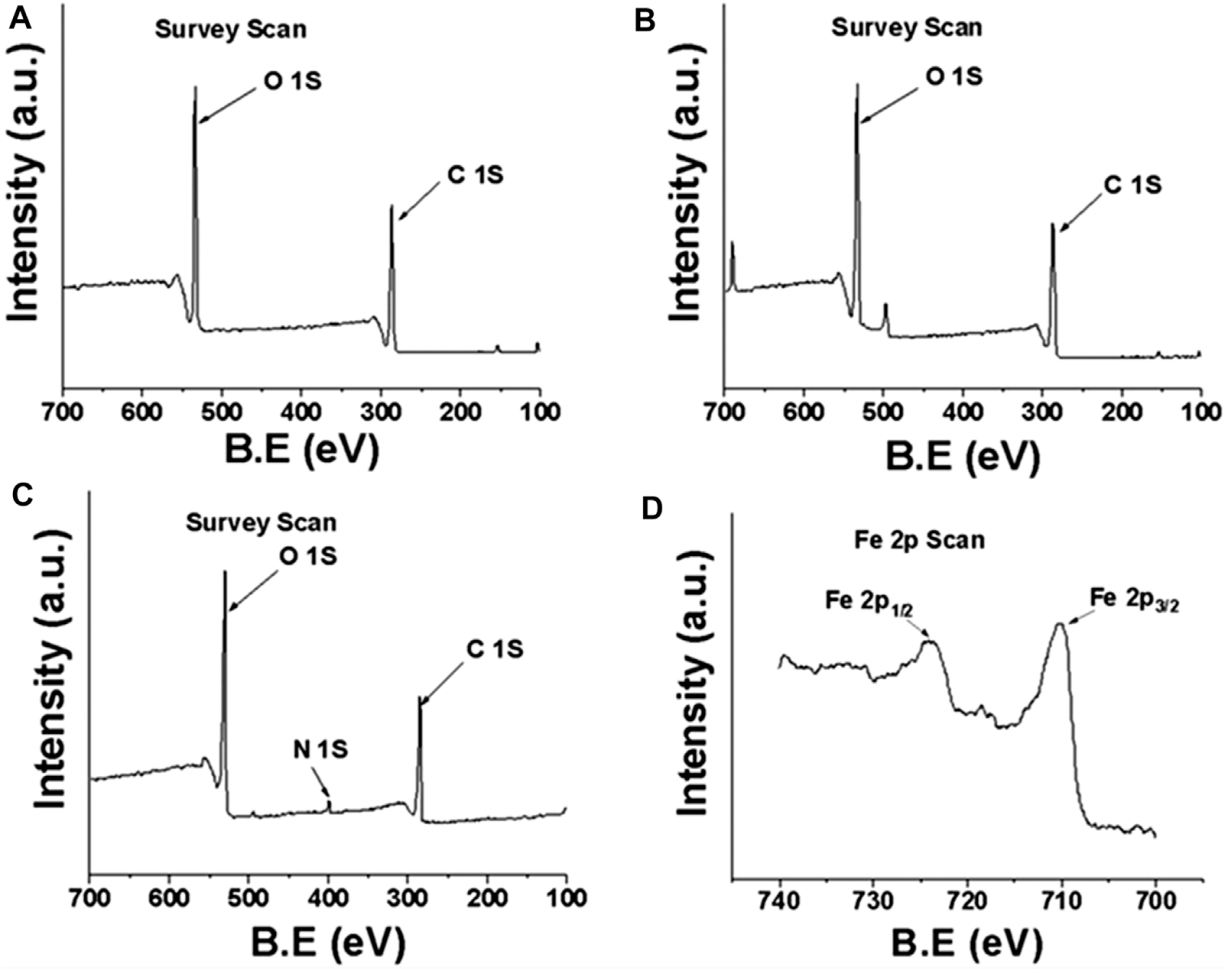
XPS survey scan: **(A)** DP, **(B)** SA-DP, **(C)** MDP@PDA, and **(D)** Fe 2p XPS spectra of MDP@PDA.

**FIGURE 5:**
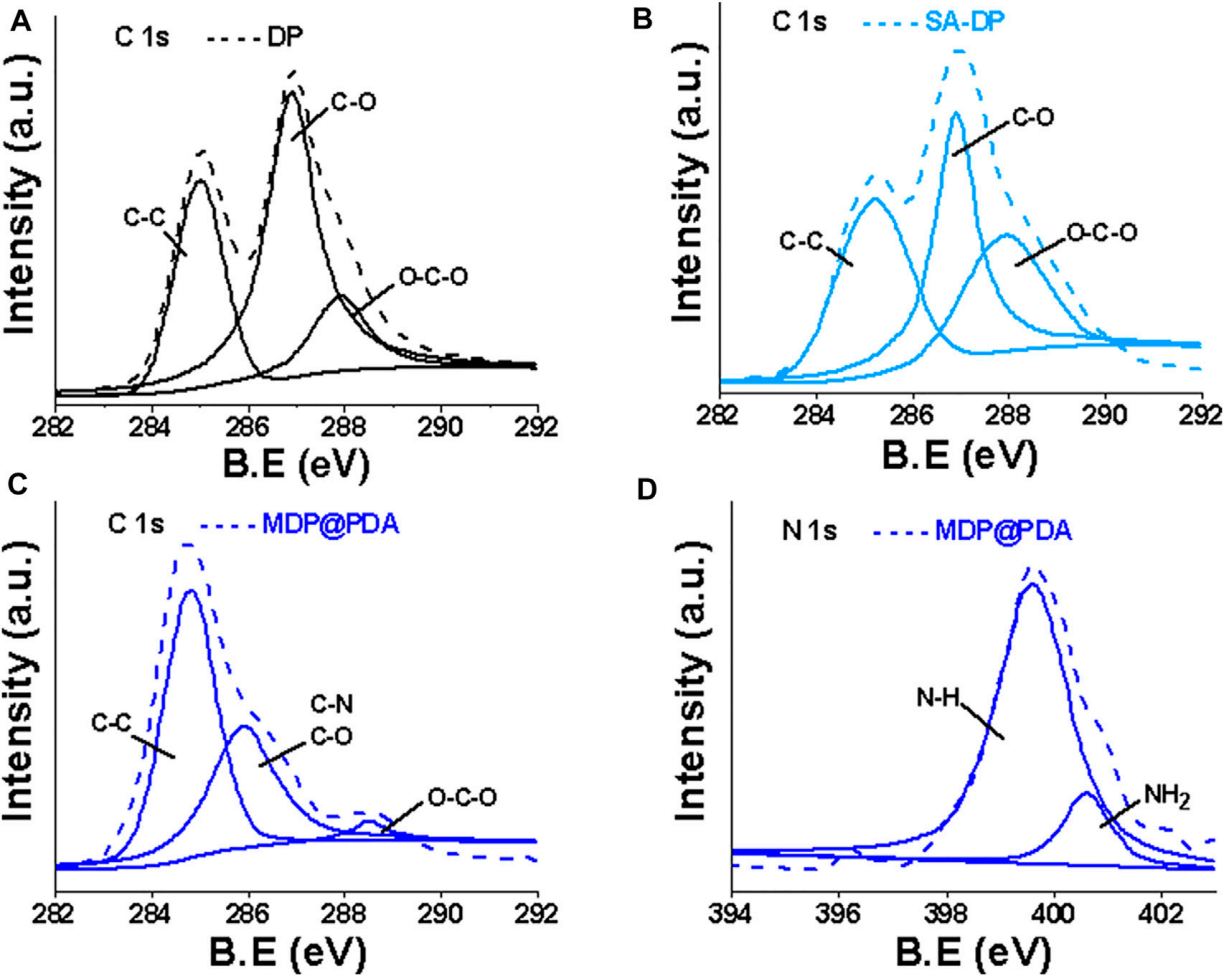
The C 1s XPS spectra of **(A**,**B)** DP and SA-DP, the C 1 s, and N 1s XPS spectra of **(C**,**D)** MDP@PDA.

XPS was used to characterize the surface elemental composition and chemical bonding information of the fibers. The XPS survey of DP fibers and SA-DP fibers showed the peak for C 1s (∼286 eV) and O 1s (∼531 eV) only. MDP@PDA fiber surface was reflected by the appearance of the N 1s (∼400 eV), Fe 2p_1/2_ (∼724.1 eV), and Fe 2p_3/2_ (∼710 eV) (Liu et al., 2016) in Figure 4. Further characterization by XPS was performed to probe the surface functionalization of the fibers as shown in Figure 5. The high-resolution C1s spectra of DP, SA-DP, and MDP@PDA fibers are shown in Figures 5A–C, components that were assigned to C-C (285.1 eV), C-O (286.9 eV), and O-C-O (288.4 eV). The high-resolution N 1s spectra of MDP@PDA fibers are shown in Figure 5D.

We observed changes in the ratio of chemical bonds C-C, C-O (mainly from cellulose structure), and O-C-O (mainly from SA modification). The N 1s region can fit three peaks at 401.3, 399.8, and 398.6 eV. The first 401.3 eV can be assigned to the amine group (R-NH_2_), which could be due to the presence of a small amount of physically self-assembled dopamine alongside polymeric dopamine. Simultaneously, the peaks at 399.8 and 398.6 eV can be attributed to PDA’s substituted amine (R-NH-R, or indolyl) and imino group (=N-R). The XPS results demonstrated the successful deposition of the PDA coating and the formation of magnetized DP fibers.

**FIGURE 6:**
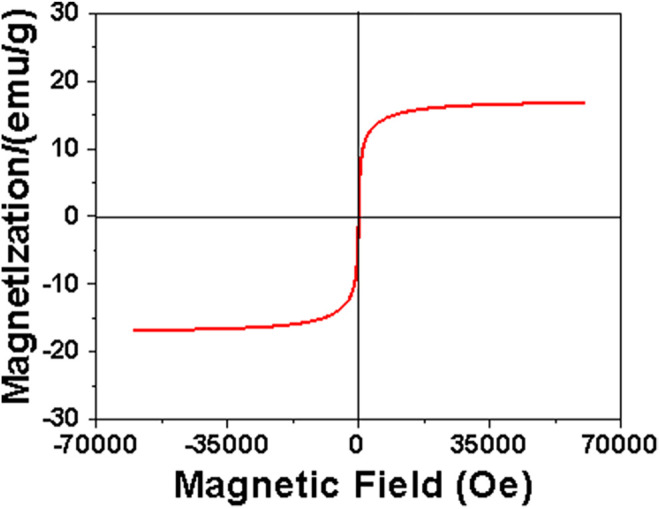
Magnetization curve of the MDP@PDA fibers.

Figure 6 presents the magnetization curve of the MDP@PDA fibers, the saturation magnetization value was 16.7 emu/g, indicating that the MDP@PDA fibers have good magnetic properties and were higher than the DP fibers (Lin et al., 2016).

**FIGURE 7:**
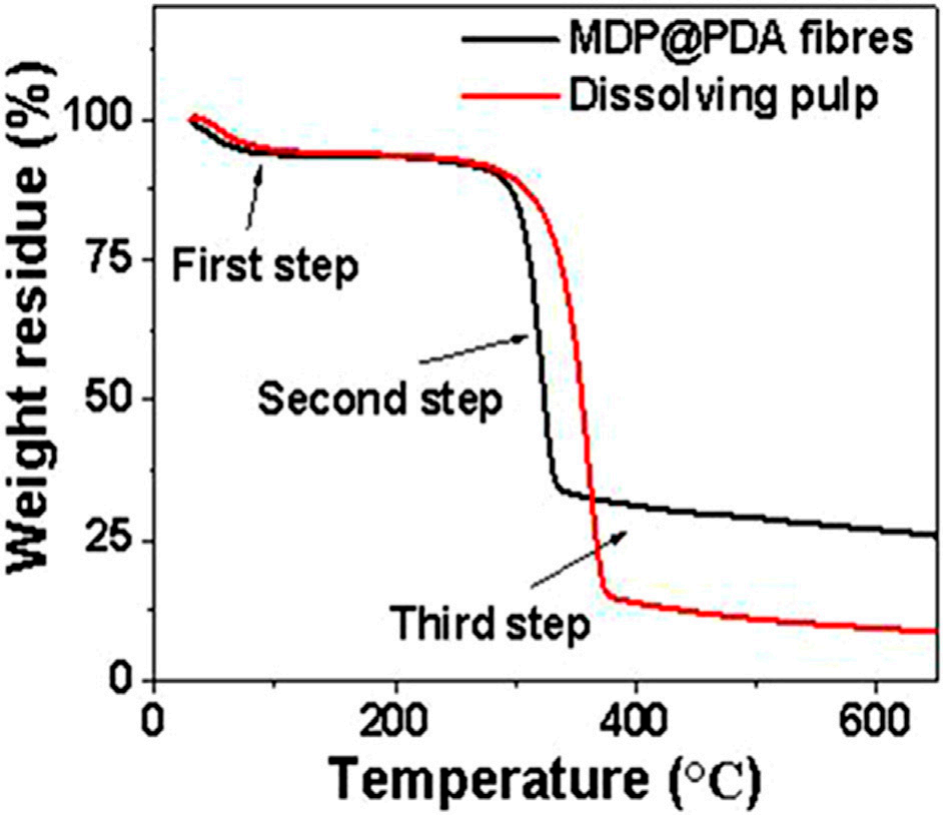
TG curve of dissolving pulp and MDP@PDA fibers.

To determine the thermal stability of cellulose and PDA, thermal analysis was performed on fiber and PDA, respectively. The thermal analysis curves of DP and MDP@PDA fibers with three steps are displayed in Figure 7. Specifically, the first step was primarily water evaporation, with a decomposition temperature of 30°C–126°C and a mass loss of approximately 6.4%. In the second step, the significant mass loss from 200°C to 400°C resulted from the rupture of cellulose polymeric chain. Obviously, the weight losses of DP and MDP@PDA fibers were approximately 86% and 61%, respectively. The third step of mass loss was assigned to the oxidative pyrolysis decomposition, the MDP mass loss reached 74% from 340°C to 600°C, and the DP mass loss reached 91% from 400°C to 600°C (Zhang et al., 2021). Compared with DP fibers, the MDP@PDA fibers retained relatively good thermal stability after chemical and magnetic modification; furthermore, it can be known from the thermal analysis curve that the content of magnetic nanoparticles on the fibers was approximately 17%.

### Adsorption Performance

**FIGURE 8:**
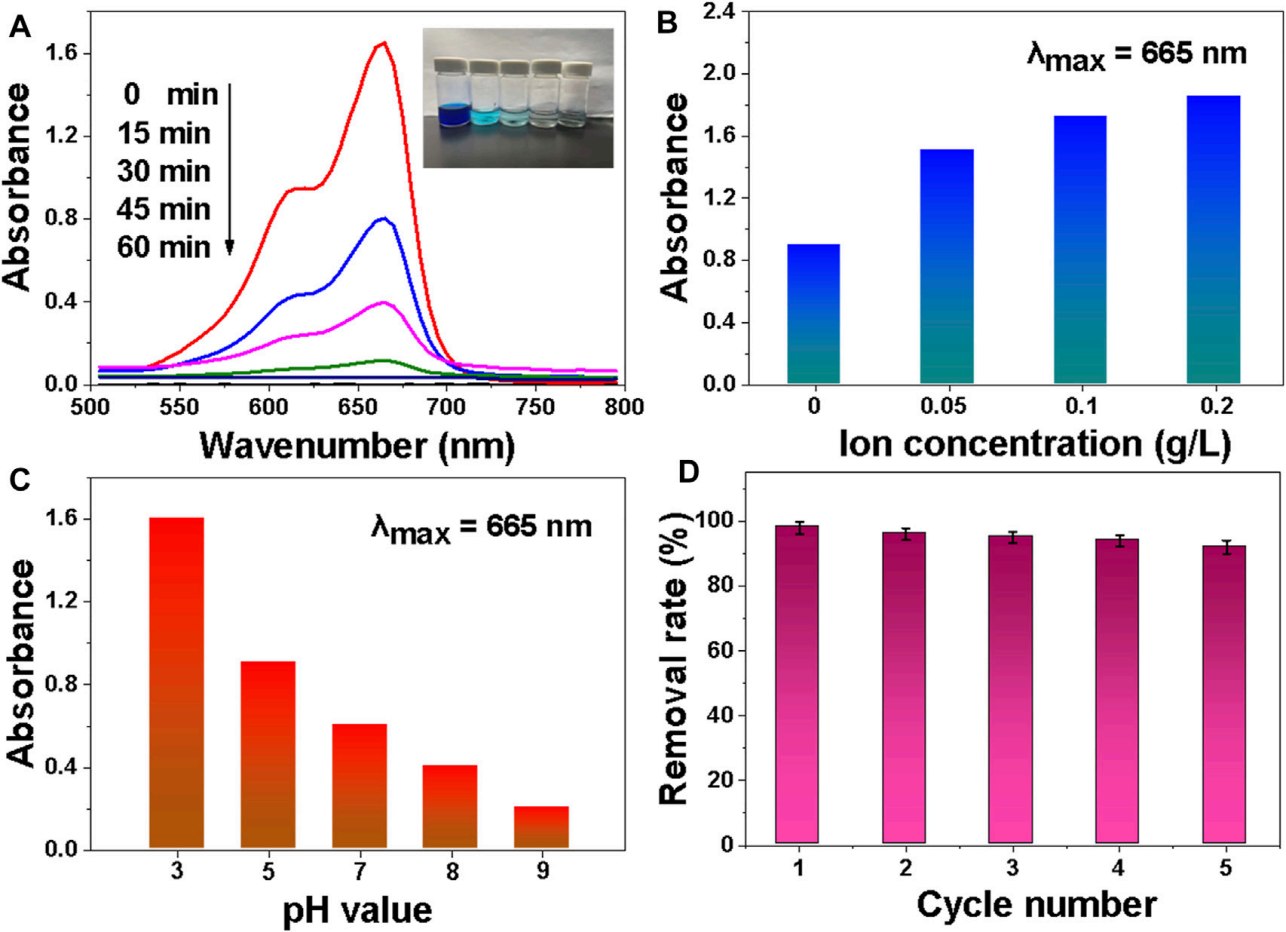
**(A)** Successive UV-vis absorption spectra of MB aqueous solution (40 mg · L^−1^) in the presence of MDP@PDA fibers. **(B)** Influence of ion concentration on MB adsorption performance. **(C)** The influence of pH value on MB adsorption performance. **(D)** The removal rate of MB with MDP@PDA fibers in different cycle numbers.

The adsorption of MB molecules by MDP@PDA fibers is a prerequisite for the catalytic reduction of MB in the NaBH_4_ system. Adsorption tests were also carried out to gain a better understanding of the catalytic reduction of MB by MDP@PDA fibers prepared as a catalytic reduction mediator and NaBH_4_ as a catalyst. Figure 8A depicts the time-dependent UV-vis spectra of the prepared MDP@PDA fiber used as an adsorbent for MB solution adsorption. The MB absorption peak began to decrease at 15 min and disappeared at 60 min, indicating that the MB in the solution was primarily absorbed by MDP@PDA fibers. Obviously, MB is the common cationic dyes (Ge et al., 2012), in order to evaluate the surface charge properly of the DP fibers and MDP@PDA fibers for the capability of MB adsorption; the zeta potential of DP fibers and MDP@PDA fibers were −6.6 ± 0.5 and −20.6 ± 2.3 mV at the pH value of 6.2, demonstrating that modified fibers exhibited more pronounced electrostatic effect on MB compared with pristine fibers as shown in Supplementary Figure S1. NaCl was used to investigate the effect of ionic strength on the adsorption performance of MB solution. As the result shown in Figure 8B, with the increase in NaCl ion concentration, the adsorption performance of the MDP@PDA fibers gradually decreased. As the previous reports (Mahmoodi et al., 2011; Metin et al., 2013; Lin Liu et al., 2015), the competitive effect of MB solution with salt ions (Na^+^ and Cl^−^) could be used to explain such results, and the competitive was evident as the NaCl concentration increase, leading to a decrease in the adsorption capacity of MDP@PDA fibers for MB. Meanwhile, this result indicated that the adsorption might be caused by electrostatic interaction between MDP@PDA fibers and dye molecules. Figure 8C shows that the pH value of the solution has a great influence on the adsorption of MB by MDP@PDA fibers. The better the adsorption effect, the higher the pH value. Because of the synergistic effects of electrostatic interaction, interaction and hydrogen bonding between PDA layers and organic dyes could be a possible adsorption mechanism. There is more electrostatic interaction when the pH value is high. The adsorbents’ recyclability was also assessed. Figure 8D shows that the MDP@PDA fibers could be recycled and reused for at least five times with a stable adsorption of more than 90%. Adsorption capacity is one of the critical indicators for evaluating adsorbents (Hokkanen et al., 2016). Table 1 shows the comparison of the comprehensive properties of absorbents for MB adsorption. Some adsorbents display better adsorption capacity (30–300 mg · g^−1^) (Fu et al., 2015; Mohammed et al., 2015; Dai et al., 2021) for MB than that of the MDP@PDA fibers (20 mg · g^−1^). However, most of the other adsorbents have limitations in magnetic properties and catalytic reduction performance by comparing with MDP@PDA fibers. Thus, these features may endow MDP@PDA fibers to have potential applications with an easy-to-operate approach in wastewater treatment fields.

**TABLE 1:** Comparison of the comprehensive properties of absorbents for MB adsorption.

Adsorbents	MB adsorption capacity (mg·g^−1^)	Magnetic	Catalytic reduction	Reference
Fe_3_O_4_@PDA-Ag	∼ 4	Yes	Yes	Cui et al. (2018)
PDA microspheres	90.7	No	No	Fu et al. (2015)
CNC–ALG	256.41	No	No	Mohammed et al. (2015)
LPMCC/LPH-0.6	51.54	No	No	Dai et al. (2021)
Cellulose/TiO_2_	0.8	No	No	Jo et al. (2017)
MDP@PDA	∼ 20	Yes	Yes	This work

### Catalytic Reduction Performance

**FIGURE 9:**
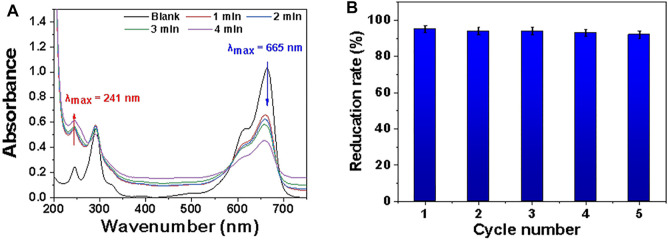
**(A)** Successive UV-vis absorption spectra UV of MB catalytic reduction process under different time conditions. The blank sample is measured after 40 mg · L^−1^ MB solution diluted 10 times. **(B)** Reduction rate of MB with MDP@PDA fibers in different cycle number.

Figure 9 shows that in the MB/NaBH_4_ system with MDP@PDA fibers as a catalyst, MB faded to completely colorless in 4 min, and while the absorbance of MB at 665 nm gradually decreased, a new absorption peak appeared at 246 nm, indicating the reduction product LMB. The addition of MDP@PDA significantly increased the rate of degradation of MB solution.

The prepared MDP@PDA fibers showed high reduction activity for MB solution (pH 3.0), although their adsorption activity for MB removal was still relatively low. The catalytic reduction of MB molecules by NaBH_4_ in the presence of Fe_3_O_4_ nanoparticles has been considered as an electrochemical mechanism, in which Fe_3_O_4_ nanoparticles act as electronic relays for MB and BH_4_^−^. Only on the surface of Fe_3_O_4_ nanoparticles can the catalytic reaction occur, where electrons are transferred from BH_4_^−^ to MB molecules. As a result, the catalytic reduction reaction requires the adsorption of MB molecules by the catalyst. The electrostatic interaction between the PDA layer and the MB molecule, as well as the π–π stacking interaction, dominate the adsorption mechanism of the prepared MDP@PDA fiber. After adding NaBH_4_ solution, BH_4_^−^ ions will gradually adsorb to the surface of MDP@PDA fibers through competitive adsorption. BH_4_^−^ ions are nucleophilic, have high electron injection capabilities, and can cathodically polarize Fe_3_O_4_ nanoparticles on the catalyst surface.

## Conclusion

In this work, MDP@PDA fibers have been prepared through a simple and easy strategy. Fe_3_O_4_ nanoparticles are grown *in situ* on the surface of the DP fibers and then self-polymerized on the fiber surface by DOPA to prepare MDP@PDA fiber. The MDP@PDA fiber showed enhanced catalytic activity based on NaBH_4_ reduction of MB as a model reaction. The chemical composition of the MDP@PDA fiber, in which the stable and immobilized Fe_3_O_4_ nanoparticles and the PDA layer have a synergistic effect, can be attributed to the excellent catalytic performance. MDP@PDA fiber has excellent recyclability because of its inherent magnetism, and it is catalytic, and adsorption efficiency does not decrease significantly after repeated use. Mussels inspired the functionalization of the PDA coating on the MDP fiber to develop multifunctional catalyst and adsorbent materials. Our research results provide a very useful and convenient method for the synthesis and adjustment of MDP@PDA fibers, which can significantly improve their catalytic and adsorption properties for organic dyes, and have huge potential applications in catalysis and wastewater treatment.

## Data Availability

The original contributions presented in the study are included in the article/Supplementary Material, further inquiries can be directed to the corresponding author.
