# Supplementary material for: Mussel-Inspired Magnetic Dissolving Pulp Fibers Toward the Adsorption and Degradation of Organic Dyes
Source: Front Chem. 2022 Mar 15;10:840133. doi: 10.3389/fchem.2022.840133 (PMC8965010; doi:10.3389/fchem.2022.840133)
Supplement: Supplementary file 1 [file DataSheet1.pdf]

Supporting Information

Mussel-Inspired Magnetic Dissolving Pulp Fibres towards the Adsorption and Degradation of Organic Dyes

Jiawei Yang[1,2], Shengchang Lu[1,3], Hui Wu[1,2], Huichao Hu[1,2], Qingxian Miao[1,2], Liulian Huang[1,2], Lihui Chen[1,2*], Yonghao Ni[1,4]

[1] College of Material Engineering, Fujian Agriculture and Forestry University, Fuzhou 350002, China;
[2] National Forestry and Grassland Administration Key Laboratory of Plant Fiber Functional Materials, Fuzhou, Fujian 350002, P. R. China;
[3] School of Forestry, Henan Agricultural University, Zhengzhou 450002, China;
[4] Limerick Pulp and Paper Centre, Department of Chemical Engineering, University of New Brunswick, NB, Canada E3B 5A3.

*** Correspondence:**
Lihui Chen
lihuichen66@126.com

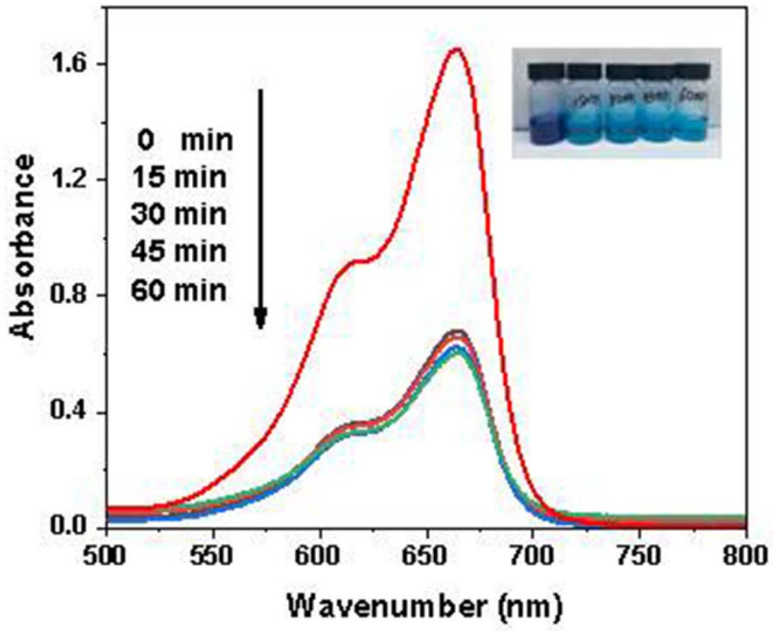

FIGURE S1 | Successive UV−vis absorption spectra of MB aqueous solution (40 mg·L^{-1}) in the presence of DP fibres.

ATR-FTIR spectra was used to compare with spectrum of MDP@PDA fibres and the MB absorbed onto MDP@PDA fibres, the result showed in **FIGURE S2**. According to the result, two prominent peaks at 1615 cm^{-1} and 1334 cm^{-1} shown in the MB adsorbed onto MDP@PDA fibres were recognized as MB with the aromatic ring vibration(Xiong et al., 2010;Liu et al., 2016), which reflected the evidence for the adsorption between MB and MDP@PDA fibres.

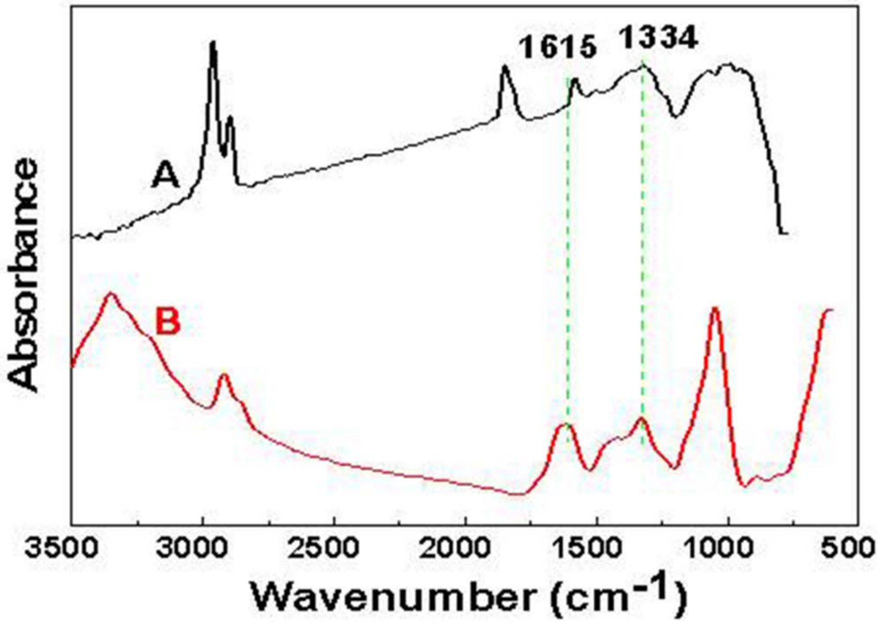

FIGURE S2 | ATR-FTIR spectra of (a) MDP@PDA, (b) MB adsorbed MDP@PDA.

References

Liu, F., Zou, H., Hu, J., Liu, H., Peng, J., Chen, Y., Lu, F., and Huo, Y. (2016). Fast removal of methylene blue from aqueous solution using porous soy protein isolate based composite beads. *Chemical Engineering Journal* 287**,** 410-418.

Xiong, L., Yang, Y., Mai, J., Sun, W., Zhang, C., Wei, D., Chen, Q., and Ni, J. (2010). Adsorption behavior of methylene blue onto titanate nanotubes. *Chemical Engineering Journal* 156**,** 313-320.
